# Pregnancy and Litter Size, But Not Lamb Sex, Affect Feed Intake and Wool Production by Merino-Type Ewes

**DOI:** 10.3390/ani9050214

**Published:** 2019-05-03

**Authors:** Manuel Ochoa Cordero, César A. Meza Herrera, Juan M. Vázquez García, Caroline A. Stewart, César A. Rosales Nieto, Ana E. Ochoa Alfaro, Ian W. Purvis, Venancio Cuevas Reyes, Héctor A. Lee Rangel, Graeme B. Martin

**Affiliations:** 1 Faculty of Agriculture and Veterinary, Autonomous University of San Luis Potosi, San Luis Potosí 78321, Mexico; manuel.ochoa@uaslp.mx (M.O.C.); manuelvazquez87@yahoo.com.mx (J.M.V.G.); ana.ochoa@uaslp.mx (A.E.O.A.); hector.lee@uaslp.mx (H.A.L.R.); 2 Regional University Unit Arid Lands, Chapingo Autonomous University Durango, Bermejillo 35230, Mexico; cmeza2020@hotmail.com; 3 School of Agriculture and Environment and UWA Institute of Agriculture, The University of Western Australia, Crawley, WA 6009, Australia; graeme.martin@uwa.edu.au; 4 CSIRO Animal Food and Health Sciences, Armidale, NSW 2350, Australia; ipurvis@csiro.au; 5 National Institute for Forestry, Agriculture and Livestock Research, Campo Experimental Valle de México, Estado de México 56250, Mexico; cuevas.venancio@gmail.com

**Keywords:** litter size, lamb sex, wool growth, Merino type sheep

## Abstract

**Simple Summary:**

We examined whether feed intake and wool production are affected by pregnancy, litter size, or lamb sex on Merino genotype ewes. We observed that feed intake was influenced during pregnancy and lactation by litter size; whereas, wool production was influenced by litter size, but not by the sex of the lambs during pregnancy.

**Abstract:**

Two experiments (Australia and Mexico) tested whether feed intake (FI) and wool production (WP) are affected by pregnancy (PRG), litter size (LZ), or lamb sex (LS) in Merino-type ewes. In Experiment-1, ewes were either not pregnant (NPR; n = 6), or carrying 1 (PR1; n = 7) or 3 (PR3; n = 11) fetuses, were studied in individual pens. NPR ewes had lower (*p* < 0.02) FI throughout PRG and lactation (LAC), except around lambing (*p* < 0.001). Following lambing, FI increased in PRG ewes (*p* < 0.001) to double the values in NPR ewes. PRG reduced WP (*p* < 0.001); in PR3, WP was lower than for both PR1 and NPR (*p* < 0.001). WP decreased during LAC and was lower in ewes rearing lambs than in NPR ewes (*p* < 0.001). Experiment-2 used 48 pregnant ewes (28 bearing singles and 20 bearing twins). Dam and lamb live weights (LW) and body condition (BC) were recorded from birth to weaning at 60 d, and dam fleece weight (DFW) was measured at weaning (12 months growth). WP was higher in ewes bearing and rearing single lambs than in ewes bearing twins (*p* < 0.001). DFW was positively (*p* < 0.01) related to LZ, dam LW, and BC, but not to changes in dam LW during LAC, or to lamb weight at birth or weaning, or LW gain, or LS. In conclusion, FI was affected during PRG and by LZ during LAC, whereas WP was influenced by LZ, but not LS, only during pregnancy.

## 1. Introduction

In sheep, wool growth depends on the availability of nutrients and, for animals grazing in the dry sub-tropics of Mexico and the Mediterranean regions of south Western Australia, nutrient availability is limited primarily by the seasonal cycle and its effects on the quantity and quality of pasture. In ewes, nutrient availability for wool growth is also determined by the physiological control systems that drive nutrients towards fetal growth during pregnancy and towards milk production after parturition [1,2,3]. Any metabolic stress therefore affects wool production [4], particularly if the diversion of body reserves to the fetus and new-born lambs leads to deficiencies in trace elements or sulphur amino acids [5,6]. The relationship between pregnancy and wool production is illustrated by the enhancement of fetal growth and birth weight when ewes are shorn in mid- or late pregnancy [7,8]. Brown et al. [9] and Rose [10] have suggested that the competition for nutrients is greater during pregnancy than lactation, although litter size was not assessed in either of these studies.

Reproducing ewes need to control body resources more carefully than non-pregnant ewes, particularly if they are bearing more than one lamb. Ewes rearing two or more lambs gain less weight during late pregnancy, lose more weight during lactation, and tend to produce less wool than ewes rearing one lamb [1,11,12]. Among the limitations of early studies in this field was the difficulty in accurately counting the number of fetuses before birth. In particular, ewes carrying three lambs, which would be the most responsive to the effects of the food supply, have rarely been studied. This limitation has been overcome by the advent of ultrasound, allowing the design of balanced, controlled, and efficient experiments.

In addition to litter size, there is the possibility that the sex of the lamb(s) will also affect wool production by the ewe. Compared to female lambs, males tend to be heavier at both birth and weaning [13,14], suggesting that they would draw more maternal resources away from wool growth. In addition, the dams of male lambs could direct more resources towards milk production because a larger fetus results in the production of more placental lactogen [15], leading to a positive relationship between birth weight and the amount of milk produced [16]. Finally, larger lambs consume more milk, thus stimulating milk production and promoting mammary gland development [17,18].

We therefore designed two experiments to test whether ewes bearing triplet fetuses and lambs will have higher feed intakes and produce less wool than ewes bearing singles. We also expected that the dams of male lambs will produce less wool than the dams of female lambs. We tested these hypotheses in experiments in Australia and Mexico, using Merino-type ewes with high potential for wool production. The findings of the two experiments are combined here because they are complementary and lead to an improvement in our understanding of the physiological mechanisms mediating the responses to the partitioning of bodily resources.

## 2. Material and Methods

### 2.1. Experiment 1

The experimental protocol was endorsed by the Animal Ethics Committee of the University of Western Australia (Approval N° 97/111) according to the recommendations of the Australian National Health and Medical Research Council.

#### 2.1.1. Location, Animals, and Management

Merino ewes (n = 100) from the Allandale Research Farm of the University of WA (31.98° S and 115.82° E) were assessed on the basis of parity, body condition, and body weight, and 24 multiparous ewes were selected for the study. The ewes were treated with intravaginal sponges (Repromap, Upjohn, MI, USA) and injected with 700 iu PMSG (Pregnant mare’s serum gonadotropin) to synchronize ovulation during the early breeding season. They were then inseminated artificially and, one month later, trans-rectal ultrasound was used for pregnancy diagnosis and estimation of litter size. Trans-abdominal ultrasound was later used to confirm the pregnancy diagnosis.

To gain experimental power by maximizing group size, and to challenge the biology by studying the extremes, we decided not to include ewes carrying only twin fetuses. The final treatment groups were therefore: Six with no fetus; 7 with a single fetus; 11 with 3 fetuses. At the time of data collection, we were not fully aware of the potential for lamb sex to affect the outcome (see Experiment 2, below), so this information was not recorded. We can only assume that lamb sex was a random factor with males and females equally represented in all treatments.

At the end of the second month of pregnancy, pregnant ewes that were diagnosed to be bearing 1 or 3 fetuses were transferred to an animal house where they were randomly allocated to individual pens where they remained for the rest of the experiment (pregnancy and lactation). In preparation for lambing, the pens were re-arranged to provide more space for the triplet-bearing ewes (2 × 2 × 1 m). Birth weight was recorded for every lamb born. Similarly to pregnant ewes, non-pregnant ewes were transferred to an animal house where they were randomly allocated to individual pens where they remained for the rest of the experiment. Throughout the period in pens, ewes were fed daily at 0830 h with a diet based on chaff, lupin grain, and minerals, as described by Masters et al. [19].

#### 2.1.2. Maternal Variables Measured

The amount of residue was assessed visually every day so the amount of feed allocated could be adjusted and an ad libitum supply could be maintained. Residues were removed daily and weighed twice weekly to quantify dry matter intake. The amount of clean wool produced by the ewes was estimated using mid-side wool patches, as described previously [20,21] approximately every 4 weeks: Baseline, early pregnancy, mid-pregnancy, late pregnancy, and lactation. In brief: On the baseline day, the skin was shorn closely with clippers on the right mid-side and tattooed with a 10 × 10 cm outline; wool was clipped from these squares, standardized for 24 h at 21 °C and 65% humidity, and weighed to provide a greasy wool weight; the wool was then washed in detergent (Teepol), ethanol, twice in solvents (Rampie Laboratories, Perth), dried in an oven, and then reweighed to produce clean wool weights (expressed as mass per 100 cm^2^). The same protocol was followed for all wool samples.

Live weight was recorded during gestation and lactation using a mobile scale with a 200 kg capacity and an accuracy of 0.05 kg.

#### 2.1.3. Progeny Variables Measured

At birth, sex and birth weight were recorded. Thereafter, live weight was recorded weekly up to weaning (age of 60 days, on average). Weight data were recorded using a mobile scale with a 200 kg capacity and an accuracy of 0.05 kg.

#### 2.1.4. Statistical Analyses

Data were analyzed with the SAS statistical package SAS version 9.3 [22]. Linear mixed model procedures and the estimation technique of restricted maximum likelihood (PROC MIXED) were used to analyze feed intake data during pregnancy, with the day of observation as the fixed effect. The number of fetuses (0, 1, or 3) was included independently as a covariate. The same approach was used for the data for clean wool production during pregnancy and lactation, with the day of pregnancy included as a fixed effect. The number of fetuses (0, 1, or 3) and dam live weight were included as covariates. Liveweight gain during lactation for lambs was fitted in a linear regression model of weight on time for each individual and the regression coefficient was estimated as a measurement of the change of weight by unit of time. Data for birth weight, liveweight gain, and weaning weight were also analyzed using PROC MIXED. Birth-rear type was included as a fixed effect. Progeny birth weight and liveweight gain were included as covariates where appropriate. Differences between pregnant and non-pregnant ewes, and among the number of fetuses (0, 1, or 3) were analyzed using PROC GLM. All 2-way interactions among the fixed effects and covariates were included in each model and non-significant (*p* > 0.05) interactions were removed from the analysis. For significant differences among mean values for treatments, within variables were analyzed using LSD of PROC GLM.

### 2.2. Experiment 2

All procedures and methods used in this study, regarding the use and care of animals, were carried-out in accordance with accepted international [23] and national [24] guidelines for animal use and care.

#### 2.2.1. Location and Animals

The study was conducted at the Facultad de Agronomía y Veterinaria, Universidad Autónoma de San Luis Potosí, Mexico (22° N) with multiparous Rambouillet ewes (n = 60) that had been born and raised in the facilities. The ewes were mated to a single Rambouillet sire of proven libido and fertility for a period of 40 days. Only pregnant ewes with similar parity and age were used and fetuses were counted at ultrasound scanning 45 days after the end of the mating period. The ewes selected for the study were carrying single (n = 28) or twin fetuses (n = 20).

#### 2.2.2. Animal Management

During pregnancy, ewes were maintained in communal pens to which they were allocated on the basis of litter size to ensure that they had sufficient space (15.0 × 15.0 m; one pen per litter). In order to avoid feed rivalry, several common feeders were provided in each pen. Ewes had free access to clean water. The diet was formulated with sorghum grain, soy and bone meal, molasses, alfalfa hay, and minerals, to meet the daily nutritional requirements for pregnancy or lactation [25]. The amount offered was adjusted every fortnight based on the mean live weight of the ewes from each experimental pen. A sample of the diet was provided to AGROLAB México S.A de C.V. and was found to be 60% digestible and to contain 12% protein, and 8.8 MJ/kg of metabolisable energy (ME). Lambs remained with their dams until they were weaned at the age of 60 days. In each pen, a creep-feeding system provided only the lambs with access to a grower diet from 10 d of age until weaning. The grower diet was based on sorghum, barley, soy meal, minerals, and vitamins, and was formulated to provide 16% protein and 70% of the total digestible nutrients [25].

#### 2.2.3. Maternal Variables Measured

The dams were shorn and their fleeces weighed on the day of weaning, so the data for wool weight reflect 12-months growth. Previously, all ewes had been shorn on the same day and managed as a single flock until the litter size was assessed, as described above. Maternal live weight and body condition score were recorded every two weeks from lambing until weaning. Body condition score was assessed using a 5-point scale, with 1 being emaciated and 5 being obese [26].

#### 2.2.4. Progeny Variables Measured

At lambing, birth weight, sex, and litter size were recorded. A total of 68 lambs were born, of which 41 were female and 27 were male. Lambs were either born as singles (14 females; 14 males) or twins (27 females; 13 males). All lambs were weighed every two weeks from birth to weaning, and the data were used to calculated the daily live weight gain. Three twin female lambs died during the experiment so were considered for the analyses of birth weight and birth type, but not the analyses of rearing type, live weight gain, or weaning weight.

#### 2.2.5. Statistical Analyses

Data were analyzed using SAS [22]. Liveweight gain during lactation for lambs was fitted in a linear regression model of weight on time for each individual and the regression coefficient was estimated as a measurement of the change of weight by unit of time. Live weight data were analyzed using linear mixed model procedures and the estimation technique of restricted maximum likelihood (PROC MIXED). Fixed effects in the model were the birth-rear type and lamb sex. Progeny birth weight and dam wool weight were included as covariates. Birth and weaning weights of lambs, as well as wool weights of dams, were analyzed using mixed models and the estimation technique of restricted maximum likelihood (PROC Mixed) and included birth-rear type and progeny sex as fixed effects. Progeny birth weight, live weight gain of dams and progeny, and body condition score of dams were included as covariates where appropriate. Differences between litter sizes and lamb sexes for live weight were analyzed using PROC GLM. All 2-way interactions among the fixed effects and covariates were included in each model and non-significant (*p* > 0.05) interactions were removed from the analysis. For significant differences among mean differences of treatments, within variables were analyzed using LSD of PROC GLM.

## 3. Results

### 3.1. Experiment 1

#### 3.1.1. Maternal Variables—Feed Intake

**Figure 1:**
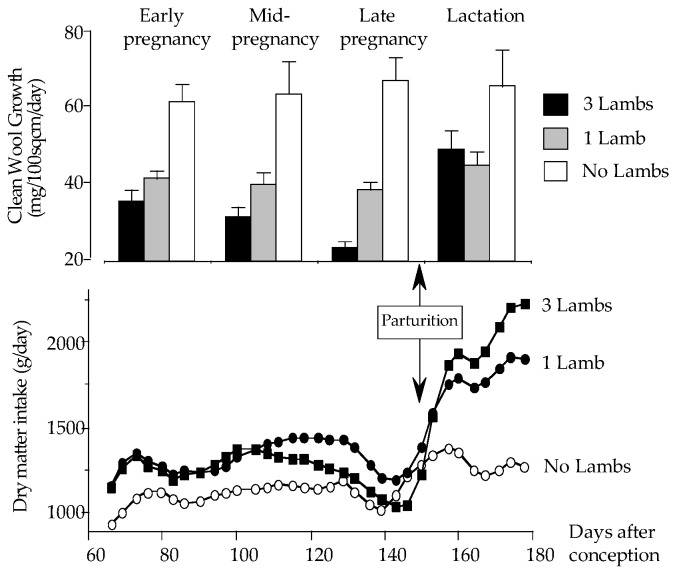
Wool growth and feed intake during pregnancy and lactation in Merino ewes bearing zero, one, or three fetuses, and then supporting zero, one, or three lambs.

Compared to pregnant ewes, non-pregnant ewes had a lower feed intake (*p* < 0.001) throughout pregnancy and lactation, except around the time of lambing (Figure 1). Feed intake was higher in the ewes bearing triplet fetuses than in those bearing single fetuses for days 115 to 135 (*p* < 0.001), but, towards the end of pregnancy, intakes decreased in all pregnant ewes and remained relatively low around the time of lambing. Following lambing, from around day 150 after conception, a robust increase in feed intake was observed in the lambing ewes (Figure 1; *p* < 0.001), and values reached were almost double those recorded for the non-pregnant ewes. The high intakes were maintained until the end of the study (*p* < 0.001). From around day 160 of pregnancy, intakes were higher in ewes rearing triplets than in ewes rearing single lambs.

#### 3.1.2. Maternal Variables—Wool Growth

In non-pregnant ewes, wool growth was constant throughout the study (Figure 1). During pregnancy, a slight reduction in wool growth was evident in single-bearing ewes, and a greater decline was evident in triplet-bearing ewes (Figure 1; *p* < 0.001). During lactation, a recovery of wool production was observed in both groups rearing lambs, with the greatest recovery observed in the ewes with triplets, although neither group produced as much wool as the non-pregnant ewes (Figure 1; *p* < 0.001).

#### 3.1.3. Maternal Variables—Live Weight

**Figure 2:**
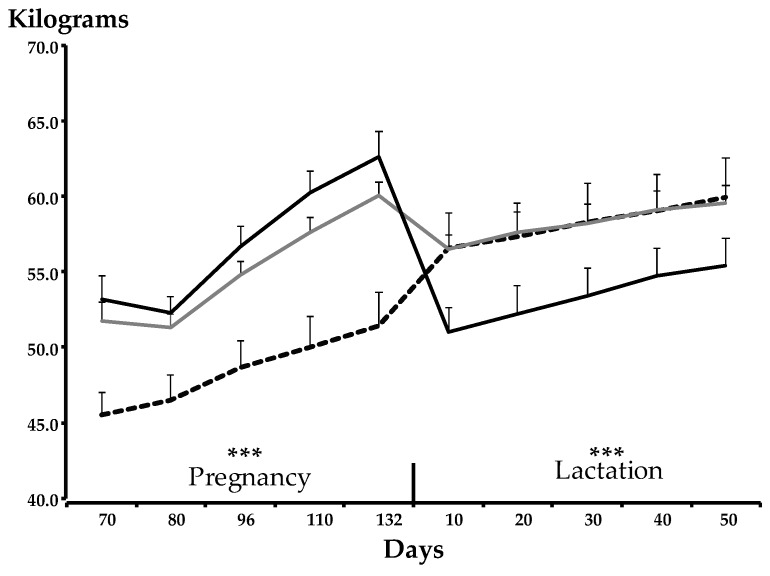
Trajectory of mean (± SEM) live weight, illustrating significant effects of pregnancy (*p* < 0.001) and lactation (*p* < 0.001), in Merino ewes bearing zero (black dotted line), one (grey solid line), or three (black solid line) fetuses, or supporting zero, one, or three lambs during lactation, in Experiment 1. Non-pregnant ewes were not lactating. *p*-values: *** *p* < 0.001.

Figure 2 shows the live weight trajectory of the ewes during the experiment; an overall effect of treatment was observed (*p* < 0.001). In the non-pregnant ewes, live weight increased from 45 to 54 kg during the period equivalent to days 70 to 132 of gestation and increased further to 58 kg by the equivalent of day 50 post-partum. In ewes bearing one fetus, live weight increased from 51 kg on day 70 of gestation to 62 kg on day 132 of gestation, and to 58 kg on day 50 post-partum. In ewes bearing three fetuses, live weight increased from 53 kg on day 70 of gestation to 63 kg on day 132 of gestation, and to 54 kg on day 50 post-partum.

#### 3.1.4. Progeny Variables—Birth Weight, Liveweight Gain, and Weaning Weight

Compared to triplet-born lambs, single-born lambs were 1 kg heavier at birth (*p* < 0.001; Table 1), grew 43% (134 g day^−1^) faster, and were 52% (8 kg) heavier at weaning (*p* < 0.001; Table 1). Live weight gain was positively related to birth weight (*p* < 0.001), such that every 1 kg increase in birth weight was associated with a 53 g day^−1^ increase in live weight gain. Weaning weight was positively related to birth weight and live weight gain (*p* < 0.001), with every 1 kg increase in birth weight being accompanied by a 3.4 kg increase in weaning weight. Moreover, for every 50 g day^−1^ increase in live weight gain, there was a 3 kg increase in weaning weight.

### 3.2. Experiment 2

#### 3.2.1. Maternal variables—Wool Production

Wool production did not differ between ewes bearing and rearing a single female lamb and ewes bearing and rearing a single male lamb (Table 2). Dams bearing and rearing singletons produced more wool than those dams bearing and rearing twins (*p* < 0.001; Table 2). Wool weight was reduced by 12% (0.7 kg) in dams rearing twins compared to those rearing singles.

#### 3.2.2. Maternal variables—Live Weight, Liveweight Change, and Body Condition Score

During lactation, dam liveweight did not differ between those rearing either singles or twins, or between those rearing either males or females (Table 2). Liveweight change in ewes did not differ between dams rearing single or twin lambs, or between dams rearing males or females (Table 2). Overall, dam body condition score was 3.1 ± 0.1 at birth and 2.7 ± 0.1 at weaning. Dam wool weight was positively related to liveweight and body condition score (*p* < 0.001), with a 0.5 kg increase in wool weight for each 10 kg increase in average live weight, and a 0.4 kg increase in wool weight for each 1-point increase in body condition score.

#### 3.2.3. Progeny Variables—Birth Weight, Liveweight Gain, and Weaning Weight

Compared to twin-born lambs, single-born lambs were 1.1 kg heavier at birth (*p* < 0.001; Table 2), grew 17% (45 g day^−1^; *p* < 0.01) faster, and were 17% (3.8 kg) heavier at weaning than twin-born lambs (*p* < 0.001; Table 2). The body condition of the dam had no effects on live weight gain from birth to weaning or on weaning weight (*p* = 0.06). Birth weight did not differ between male and female lambs (*p* > 0.05), but male lambs grew 10% faster and were 8% heavier at weaning than female lambs (*p* < 0.05; Table 2). Live weight gain was positively related to birth weight (*p* < 0.001), such that every 1 kg increase in birth weight was associated with a 30 g day^−1^ increase in live weight gain. Weaning weight was positively related to both birth weight and lamb live weight gain (*p* < 0.001). Every 1 kg increase in birth weight was associated with a 2.7 kg increase in weaning weight and every 50 g day^−1^ increase in live weight gain was associated with a 3.3 kg increase in weaning weight.

## 4. Discussion

Our results show that feed intake was higher and wool production was significantly lower in ewes bearing triplet fetuses, compared to ewes bearing a single fetus, supporting our first hypothesis. However, wool production from dams with male or female lambs did not differ, so we rejected our second hypothesis. A substantial increase in feed intake, and subsequent live weight, after mid-pregnancy, has been interpreted as reflecting the positive relationship between feed intake and the increase in accumulated mass of the feto-placental unit(s) [27,28,29], with the effect being most evident in ewes bearing multiple fetuses ([30], present study). However, it is important to remember that the accelerated accumulation of resources, and the redistribution towards feto-placental unit(s) is not a passive process (the “sink” concept), but a controlled process that has evolved to ensure metabolic support for fetal growth and development [31,32].

On the other hand, in the period leading up to lambing, the dramatic decline in feed intake that we observed in the pregnant ewes was most likely caused in part by the re-organization of the pens, because there was a similar, albeit smaller, decline in the non-pregnant ewes. Nevertheless, our observations support those of Bermudez et al. [33], and it seems likely that the decline in feed intake as lambing approaches reflects physical compression of the rumen by the rapidly expanding uterus [34,35]. This hypothesis is consistent with the greater decline in feed intake in triplet-bearing ewes compared to single-bearing ewes in the present study, and the rapid increase in feed intake following lambing, as observed by Hadjipieris and Holmes [36] and Lee and Atkins [27].

Pregnancy reduced wool growth in ewes when compared to non-pregnant ewes being fed the same diet. Among pregnant ewes, those bearing multiple fetuses were more affected than those bearing a single fetus, confirming the observations of Lee and Atkins [27] and Waters et al. [29]. Wool growth recovers after lambing, even as lactation progresses, but wool production fails to attain the levels seen in non-pregnant ewes [1,11,12,37]. As with the effects of pregnancy and litter size on feed intake, the effects on wool production reflect the physiological control processes that actively direct nutrients to the fetus rather than to the wool follicles [9,37], thus ensuring that the nutritional demands for fetal development and milk production are met before those for wool production. These processes make it difficult to ensure the maintenance of wool production during pregnancy and lactation simply by improving nutrition [3,12].

In contrast to our expectations, wool production was not lower in dams bearing male lambs than in those bearing female lambs. During pregnancy, nutrient partitioning is thought to favor male lambs because they grow faster in utero [13], and are heavier at birth and weaning, apparently because the somatotrophic axis is more active in male fetuses than female fetuses [14,38,39]. Moreover, heavier lambs stimulate milk production by removing more milk from their dams than lighter lambs [18,40], thus explaining differences in growth between single- and twin-born lambs, and between male and female lambs [41,42]. Theoretically, the combination of these effects should direct nutrients away from wool production, yet we detected no significant effect of progeny sex on dam wool production. The dams in the present study were probably meeting their nutritional requirements [26], thus avoiding competition for nutrients between the wool, fetus, and milk. The lack of significant differences between the sex of lambs in dam wool production and birth weight suggests that the sample size used in this experiment might have been too small. Moreover, an effect of lamb sex on dam wool production might become more evident with dietary restriction during pregnancy and lactation [43].

For the Rambouillet dams in the current study, lamb birth weights and wool production were within the normal range for this breed [44,45]. However, we also observed that dam wool weight was not related to lamb birth weight, lamb live weight gain, lamb weaning weight, or maternal live weight change during lactation. There is little information about the effect of progeny weight, at birth or weaning, on maternal wool production. However, for the lambs, the amount of wool they produce after reaching adulthood is positively correlated with their birth and weaning weights [46], perhaps reflecting the positive relationship between body weight and wool production, with heavy animals producing more wool than light animals [47,48]. Similarly, during lactation, we observed positive relationships between dam live weight and dam wool production, and between dam body condition and dam wool production, extending on previous observations [9,10,12,20,37,41,49]. These outcomes support the contention that genetic selection for an increase in live weight has a positive effect on wool production [47].

Lambs born as singletons were heavier at birth, grew faster, and were heavier at weaning than lambs born as twins or triplets. These observations agree with differences between single and twin-born lambs in birth weight and growth that have been reported previously [13,14]. Moreover, we observed a positive correlation between birth weight and weaning weight, and between birth weight and live weight change, confirming our previous observations [14,39]. The difference between male and female lambs was 0.3 kg, and not significant, whereas previous studies have reported significant differences in this trait [13,14].

## 5. Conclusions

In summary, in Merino-type ewes, feed intake and the amount of wool produced are affected by the number of fetuses they carry during pregnancy and by the number of lambs they subsequently rear during lactation. Feed intake is related to ewe live weight whereas wool production is related to ewe live weight and body condition score, but the sex of the lamb had no discernible effects. Pregnancy reduces wool production and this management challenge is especially important for ewes bearing multiple fetuses.

## Figures and Tables

**Table 1:** Birth weight, weaning weight, and daily liveweight gain in Merino lambs in Experiment 1. Values are represented by mean ± SEM.

	Birth Weight (kg)	Liveweight Gain (g day^−1^)	Weaning Weight (kg)
**Birth Type**			
Single	3.8 ± 0.45 ***		
Triples	2.7 ± 0.15		
**Birth-Rear Type**			
Single (1-1)		235 ± 10.1 ***	16.9 ± 0.73 ***
Triples (3-3)		101 ± 14.6	8.8 ± 0.89

*** *p* ≤ 0.001.

**Table 2:** Live weight change (LWC), body condition (BC), and wool weight (FWT) in Rambouillet ewes during lactation in Experiment 2. Growth of their lambs from birth (birth weight (BWT)) to weaning (weaning weight (WWT)), allowing measurement of live weight gain (LWG), in lambs born and raised as singles (1-1), born and raised as twins (2-2), or born as female or male. Values are represented by mean ± SEM.

Variable		BWT (kg)	LWG (gr)	WWT (kg)	LWC (gr)	BC	FWT (kg)
	Progeny Variables				Maternal Variables		
**Birth Type**	n	Mean ± SEM					
Single	28	5.5 ± 0.11 ***				3.3 ± 0.2	
Twin	40	4.4 ± 0.09				3 ± 0.2	
**Lamb Sex**	n						
Female	41	4.8 ± 0.11	237 ± 9.4 *	19 ± 0.6 *	−60.4 ± 15	3.2 ± 0.2	5 ± 0.1
Male	27	5.1 ± 0.18	263 ± 8.1	20.7 ± 0.6	−55 ± 13	3.1 ± 0.2	5.1 ± 0.2
**Birth Type-Rear Type**							
Single-Single	28		273 ± 7.0 **	21.9 ± 0.5 ***	−73 ± 16	2.9 ± 0.2	5.4 ± 0.1 **
Twin-Twin	37		228 ± 9.0	18.1 ± 0.6	−48 ± 13	2.9 ± 0.2	4.7 ± 0.1

*p*-values: * *p* ≤ 0.05; ** *p* ≤ 0.01; *** *p* ≤ 0.001. For birth–rear type, data are combined for male and female lambs; for sex of lamb, data are combined for single and twin birth–rear types.
